# Simultaneous cell disruption and semi-quantitative activity assays for high-throughput screening of thermostable L-asparaginases

**DOI:** 10.1038/s41598-018-26241-7

**Published:** 2018-05-21

**Authors:** Xu Li, Xian Zhang, Shuqin Xu, Hengwei Zhang, Meijuan Xu, Taowei Yang, Li Wang, Haifeng Qian, Huiling Zhang, Haitian Fang, Tolbert Osire, Zhiming Rao, Shangtian Yang

**Affiliations:** 1 The Key Laboratory of Industrial Biotechnology, Ministry of Education, School of Biotechnology, Jiangnan University, Wuxi, 214122 China; 2 School of Food Science and Technology, Jiangnan University, Wuxi, 214122 China; 3 School of Agriculture Ningxia University, Yinchuan, 750021 China; 4 Department of Chemical and Biomolecular Engineering, The Ohio State University, Columbus, OH 43210 USA

## Abstract

L-asparaginase, which catalyses the hydrolysis of L-asparagine to L-aspartate, has attracted the attention of researchers due to its expanded applications in medicine and the food industry. In this study, a novel thermostable L-asparaginase from *Pyrococcus yayanosii* CH1 was cloned and over-expressed in *Bacillus subtilis* 168. To obtain thermostable L-asparaginase mutants with higher activity, a robust high-throughput screening process was developed specifically for thermophilic enzymes. In this process, cell disruption and enzyme activity assays are simultaneously performed in 96-deep well plates. By combining error-prone PCR and screening, six brilliant positive variants and four key amino acid residue mutations were identified. Combined mutation of the four residues showed relatively high specific activity (3108 U/mg) that was 2.1 times greater than that of the wild-type enzyme. Fermentation with the mutant strain in a 5-L fermenter yielded L-asparaginase activity of 2168 U/mL.

## Introduction

L-asparaginase (EC 3.5.1.1), which catalyses the conversion of L-asparagine to L-aspartate and ammonia, has been identified in various organisms, including animals, plants, yeast, fungi, bacteria, and archaea^1–3^. L-asparaginase has high commercial value due to its vast applications in the food and pharmaceutical industries^2,4^. In clinical applications, L-asparaginase is used to treat lymphoblastic leukaemia, as it removes the L-asparaginate required for the survival of lymphoblastic leukaemia cells^5^. To date, L-asparaginases from *Erwinia chrysanthemi* and *Escherichia coli* have been used as effective antitumour drugs for the treatment of paediatric acute lymphoblastic leukaemia^5–7^. In the food industry, L-asparaginase is used to reduce L-asparagine, which is the precursor of carcinogenic acrylamide^8,9^. During this process, L-asparaginase is treated at a high temperature together with raw food materials or is sometimes blanched in hot water^10–12^; therefore, the enzyme should be capable of withstanding high temperatures. However, the low activity and instability of L-asparaginase at high temperatures have restricted its application in the food industry.

To effectively apply L-asparaginase industrially, some researchers are using protein engineering methods obtain L-asparaginase mutation with ideal properties. Long *et al*. identified some key amino acid residues adjacent to the catalytic cavity of L-asparaginase and improved its thermostability and catalytic efficiency via mutagenesis of these residues^13^. By employing a genetic algorithm in tandem with flexibility studies using molecular dynamics, Offman *et al*. engineered L-asparaginase with improved activity and resistance to proteolytic cleavage for ideal clinical applications^14^. Furthermore, Kotzia and Labrou constructed a mutagenesis library via a staggered extension process using L-asparaginase genes from *E. carotovora* and *E. chrysanthemi* and then created a new enzyme variant with improved thermal stability via directed evolution^15^. Regardless of the approach used for mutant construction, an appropriate screening method can simplify the identification process. However, few reports have applied high-throughput screening for L-asparaginase. Instead, some reports have used pH indicator dye-based plate assays to screen for L-asparaginase-producing microorganisms^16–18^. In these methods, the pH increases due to the generation of ammonia when strains secrete L-asparaginase, and a differentiable zone form around the strain on the L-asparagine plate. These methods have been used to screen for extracellular L-asparaginase-producing microbes^17^. Because the working temperature for thermophile L-asparaginase is greater than 60 °C^19^, strains have difficulty growing at that temperature. Thus, the discordant temperature limits the application of these methods for the screening of strains with thermophilic L-asparaginases.

Furthermore, L-asparaginase genes have been widely expressed in many microbial host systems, and a variety of strategies have been used to improve L-asparaginase yields in these strains^20^. Meena *et al*. expressed the *Streptomyces griseus* L-asparaginase gene in *E. coli* M15, with an enzyme activity of 123 U/mL^3^. Ferrara *et al*. used *Pichia pastoris* as a host to express the *Saccharomyces cerevisiae* L-asparaginase gene under the control of the AOX1 promoter in a 2-L instrumented bioreactor, with a resultant yield of 85.6 U/mL^21^. Feng *et al*. over-expressed the *Bacillus subtilis* L-asparaginase gene in *Bacillus subtilis* with the highest yield ever reported in a food-grade host of 407.6 U/mL in a 3-L fermenter after signal peptide screening, promoter mutation, and N-terminal deletion^22^. Amardeep *et al*. cloned the *E. coli* L-asparaginase genes and over-expressed them in *E. coli* DE3 under the inducible T7 *lac* promoter. Using the DO-stat fermentation strategy in a 2-L bioreactor, the yield was 870 U/mL at an OD_600_ of 90^23^, which was the highest yield ever reported.

In this study, a new thermostable L-asparaginase from *Pyrococcus yayanosii* CH1 was characterized and expressed in *B. subtilis* 168. To identify thermophilic L- asparaginase mutants with higher activity, a robust high-throughput screening method was developed, and some positive variants were identified. Moreover, through medium optimization and the pH-stat fermentation strategy, a maximum volumetric yield of 2168 U/mL of L-asparaginase was obtained after 36 h of fermentation in a 5-L fermenter.

## Materials and Methods

### Strains, plasmids, and chemicals

*B. subtilis* 168 was used as the host strain for gene cloning and expression. The shuttle expression plasmid pMA5 was used for the expression and mutagenesis studies. All strains and plasmids were preserved in our laboratory. The restriction enzymes, PrimeSTAR® HS DNA Polymerase and T4 DNA ligase were purchased from TaKaRa Bio Co. (Dalian, China), and the Mini Plasmid Rapid Isolation Kit, DNA Extraction Kit, and Mini DNA Rapid Purification Kit were obtained from Sangon Biotech Co., Ltd. (Shanghai, China). A HisTrap^TM^ HP column was purchased from GE Healthcare, Inc. (Little Chalfont, U.K.). All other high-grade chemicals were commercially sourced.

### Construction of recombinant strains and the mutagenesis library

The *P. yayanosii* CH1 L-asparaginase (NCBI accession number: WP_013906452) gene (*pyasnase*) was synthesized in the pUCk plasmid by Sangon Biotech (Shanghai, China) after its sequence (Supplementary Table 1) was optimized for *Bacillus subtilis*. The L-asparaginase gene (*pyasnase*) was amplified by PCR with the primer pair F1 and R1 (Table 1). The PCR amplicon and pMA5 plasmid were digested with *Bam*HI and *Mlu*I, and the two digested fragments were ligated using the T4 ligase to form the recombinant plasmid pMA5-*pyasnase*.

**Table 1:** Primers used in this study. The underlined text indicates the restriction sites. The bold type of R1 indicates the His-tag gene. The sites of the variations are shown in lowercase letters.

Primers	Sequences 5′-3′
F1	CGGGATCCATGAGACTGCTGATCCTGG
R1	GCACGCGTTTA**GTGGTGGTGGTGGTGGTG**CGCGGATTTCCCAATTTCG
F2: S17G	AGTGTGCCTggcGAAGAGGGATACGAATCATCACTGT
R2:S17G	TCTTCgccAGGCACACTTGCGATTGTTCCTCC
F3:A90S	CGCTTACACAagtTCGATGCTTAGCTTTATGGTGAGA
R3: A90S	TCGAactTGTGTAAGCGAGCGTGTCTGTACCG
F4: R156S	CAAGGTCagtGCAGTTGGTCTTAACGCCTTTC
R4: R156S	CAACTGCactGACCTTGCTTACTCTCACTCCGA
F5: K272A	CTTGACCgccTACAAAGTCGGCCGGAAAGCGT
R5: K272A	CTTTGTAggcGGTCAAGTCAACGCCGTCATAT

The mutagenesis library was constructed using the method described by Roberts and Zhang^24,25^. To construct the mutagenesis library, error-prone PCR (epPCR) was performed with the same primer set used for L-asparaginase gene amplification with the Genemorph II Random Mutagenesis Kit (Stratagene, La Jolla, CA, USA). The recombinant plasmid pMA5-*pyasnase* was used as a template for the first round of epPCR, and the three bright positive mutation-coding genes from the first round were used as templates for the second round of epPCR. Then, the epPCR amplicon was ligated into the pMA5 plasmid at the *Bam*HI and *Mlu*I sites.

Site-directed mutagenesis of *pyasnase* was accomplished by overlap-extension PCR. Mutations were introduced using the primers listed in Table 1. The mutants were also ligated into the pMA5 plasmid at the *Bam*HI and *Mlu*I sites. All recombinant plasmids were sequenced by Sangon Biotech.

### Screening for high-activity variants

The recombinant plasmid with the epPCR-mutated gene was transformed into the expression strain *B. subtilis* 168 on Luria-Bertani (LB) solid medium for 12 h. Single colonies were incubated in preheated 96-deep well plates containing a 0.5 mL reaction mixture (25 mM L-asparagine and 50 mM Tris-HCl, pH 8.0) at 95 °C for 10 min. One hundred µL of 15% trichloroacetic acid (TCA) was used to stop the reaction. The chromogenic reaction was conducted by adding 10 µL of Nessler’s reagent, and the reaction mixture was incubated for 3 min at room temperature. Afterwards, 200 µL of supernatant was collected from each 96-well plate after centrifugation at 20,000 × g for 10 mins, and the absorbance was measured at 450 nm using a microplate reader (epoch2, Biotek, USA). *B. subtilis* 168/pMA5-*pyasnase* (wild-type) was used as a control.

### L-asparaginase expression, purification and activity assay

The recombinant plasmid was transformed into the expression strain *B. subtilis* 168 and the recombinant strain was cultured at 37 °C and 200 rpm for 12 h as a seed solution in LB medium containing 20 μg/mL of kanamycin. One mL of seed solution was added to 100 mL of basal medium and cultured under the same conditions. After 24 h of cultivation, the cells were harvested by centrifugation at 10,000 × g for 10 min at 4 °C. The supernatant was used to conduct an activity assay, and the cells were re-suspended and washed twice in lysis buffer (50 mM Tris-HCl buffer, pH 8.0). Then, 6 mg/mL of lysozyme was added to the lysis buffer containing the cells for 2 h, followed by ultrasonication at 20 MHz with 65% amplitude for 30 min. The lysate was centrifuged at 20,000 × g for 10 min, and the clear supernatant was used as the crude enzyme source.

Ni^2+^-affinity chromatography and an AKTA purifier system (GE Healthcare, Sweden) were used to purify the crude enzyme. The crude enzyme was loaded onto a 1-mL HisTrap^TM^ HP column with Binding Buffer (0.02 M Tris-HCl buffer and 0.5 M NaCl, pH 7.4) with a 0.5 mL/min loading rate. L-asparaginase was eluted at 1 mL/min with a linear gradient of imidazole concentrations ranging from 0 to 0.5 M. Then, the purified enzyme was dialyzed with PB buffer (0.05 M, pH 7.0) or Tris-HCl buffer (0.05 M, pH 7.0) to remove imidazole and recycled for SDS-PAGE analysis.

The L-asparaginase activity assay was conducted at 95 °C as described by Long and Zuo *et al*.^13,19^. The reaction mixture (1 mL) containing L-asparagine (25 mM) and Tris-HCl (50 mM, pH 8.0) was preheated at 95 °C. Then, 100 µL of enzyme solution was added and reacted with the substrate for 10 min. One hundred µL of 15% trichloroacetic acid (TCA) was used to stop the reaction. The reaction mixture was centrifuged at 20,000 × g, mixed gently with 200 µL of the clear supernatant, 4.8 mL of deionized water and 200 µL of Nessler’s reagent, and the amount of ammonia released was measured. All measurements were performed spectrophotometrically at 450 nm. Since L-asparagine is sensitive and prone to high-temperature hydrolysis, interference by asparagine self-hydrolysis should be prevented. Thus, TCA and enzyme solution were successively added to the reaction mixture and were used as a blank during the spectrophotometric enzyme activity assays. One unit of L-asparaginase activity was defined as the amount of enzyme required to release 1 µmol of ammonia per minute under assay conditions. The protein concentrations were determined at 25 °C using a Bradford protein assay kit.

### Determination of enzyme properties

The optimum temperature of L-asparaginase was examined using 50 mM Tris-HCl buffer (pH 8.0) with temperatures ranging from 40 to 100 °C. The optimum pH was measured by assaying the enzyme activity at various pH values (0.05 M acetate buffer, pH 4.0–6.0; 0.05 M phosphate buffer, pH 6.0–7.0; 0.05 M Tris-HCl buffer, pH 7.0–9.0; and 0.05 M glycine-NaOH buffer, pH 9.0–10.0) at 95 °C.

The thermal stability of L-asparaginase was determined by incubating the enzyme in Tris-HCl buffer (50 mM, pH 7.0) for 15–120 min at 70, 75, 80, 85, 90, and 95 °C. After incubation, the protein was refolded on ice for 15 min, and the residual enzyme activity was measured at 95 °C and pH 8.0. The enzyme stability at the storage temperatures (−20, 4, 20, and 37 °C) was determined as described above. Additionally, the pH stability of L-asparaginase was determined by incubation in different buffers with pH values ranging from 4–10 at 4 °C for 3–30 h. The stability of L-asparaginase in urea was determined by incubating the enzyme in gradient concentrations (0–8 M) at 4 °C for 6 h. The residual enzyme activity in the pH and urea stability assays was measured using the method described for the thermal stability assay.

The effects of various metal ions (Mg^2+^, Ca^2+^, Zn^2+^, Co^2+^, Mn^2+^, Ba^2+^, Ni^2+^, and Cu^2+^) on the enzyme activity were also examined using a reaction mixture supplemented with a 1 mM concentration of these various cations. An L-asparaginase mixture without any added metal ions was used as the control.

The Michaelis constant (K_m_) and maximal velocity (V_max_) were determined in 50 mM Tris-HCl buffer (pH 8.0) at 95 °C by changing the concentration of the substrate (L-asparagine) in the range of 0.05–2.0 mM. Michaelis-Menten plots were used to calculate these values. All of the assays were performed in triplicate.

### Bioinformatics analysis

A circular dichroism spectrometer (MOS-450 model, French Biologic Company, France) was used to study the secondary structure of 0.1 g/L of purified enzyme at 25 °C. The sample spectra were scanned in the far UV range (190–250 nm) at a rate of 30 nm/min, with PB buffer (0.05 M, pH 7.6) used as a blank. The online server DICHROWEB (http://dichroweb.cryst.bbk.ac.uk/html/process.shtml) was used to predict the rates of different secondary structures in the enzyme.

The molecular weight, theoretical pI, and instability index were predicted using ProtParam (http://web.expasy.org/protparam/). The structural model of L-asparaginase was acquired by homology modelling using SWISS-MODEL (http://swissmodel.expasy.org/). The quality of the generated model was checked using the VERIFY_3D Server (http://services.mbi.ucla.edu/Verify_3D/)^26,27^. Program PYMOL was used to analyse variation in the molecular tertiary structure^28^.

### Medium optimization and fermentation

One-factor-at-a-time and orthogonal array design methods were used to optimize the medium. The basal medium (pH 7.0) contained (g/L): sucrose, 35; tryptone, 15; urea, 0.8; corn steep liquor, 12; K_2_HPO_4_, 2.612; KH_2_PO_4_, 2.041; MgSO_4_·7H_2_O, 1.845; NaCl, 5; and L-Asn, 1. The optimized medium (pH 7.0) contained (g/L): glycerine, 47; yeast, 35; NH_4_Cl, 1.5; corn steep liquor, 15; K_2_HPO_4_, 2.612; KH_2_PO_4_, 2.041; MgSO_4_·7H_2_O, 1.845; NaCl, 5; and L-Asn, 1. The feed medium for fed-batch cultivation contained (g/L): glycerine, 500, and yeast, 75.

Fed-batch cultivation was conducted in a 5-L computer-controlled fermenter (Bao Xing, China) with 2 L of optimized medium. The expression strain was cultured at 37 °C with shaking at 200 rpm in 100 mL of LB medium for 12 h as the seed solution. The aeration rate, agitation speed, and temperature were 4.0 vvm, 600 rpm, and 37 °C, respectively. The pH was maintained at 7.0 using NH_4_OH (30%v/v) and the feed medium. NH_4_OH was automatically dosed whenever the pH dropped below 7.0, whereas the feed medium was added automatically by the acid-supplying pump when the pH rose above 7.0.

## Results and Discussion

### Sequence analysis and construction of the recombinant plasmid

*Pyasnase* from *P. yayanosii* CH1 is a 987-bp gene encoding 328 amino acids. BLAST analysis showed 65% similarity between the *P. yayanosii* CH1 L-asparaginase and L-asparaginase I from *Thermococcus sp*. EP1 (WP_055283058.1). The protein had a predicted molecular mass of 36.1 kDa and a theoretical pI of 5.16. The instability index of the enzyme was computed to be 29.80 and was classified as generally stable^29^. The L-asparaginase gene was ligated into the pMA5 vector between the *Bam*HI and *Mlu*I restriction sites with gene transcription controlled by the HpaII promoter, resulting in the recombinant vector pMA5-*pyasnase*.

### L-asparaginase expression and purification

To obtain an L-asparaginase recombinant strain suitable for food industry applications, the food-grade strain *B. subtilis*168 was used as a host strain to express L-asparaginase. After 24 h of cultivation with basal medium, the extracellular and intracellular L-asparaginase activities were 23.31 and 65.72 U/mL, respectively.

Ni^2+^-affinity chromatography was used to purify the protein. A 12% SDS-PAGE analysis (Supplementary Fig. 1) indicated that the protein was purified and that the molecular mass was the same as previously predicted. The specific activity of the purified enzyme was 1483.81 U/mg (Table 2).

**Table 2:** Purification of recombinant L-asparaginase.

Purification step	Volume (mL)	Total protein (mg)	Total activity (U)	Specific activity (U/mg)	Purification fold	Yield (%)
Crude enzyme	5	23.87 ± 1.3	699.75 ± 3.3	29.31 ± 1.84	1	100
Ni^2+^-affinity chromatography	3	0.2873 ± 0.01	426.3 ± 2.8	1483.81 ± 63.61	50.62 ± 5.6	60.92 ± 0.7

### Enzyme characterization

**Figure 1:**
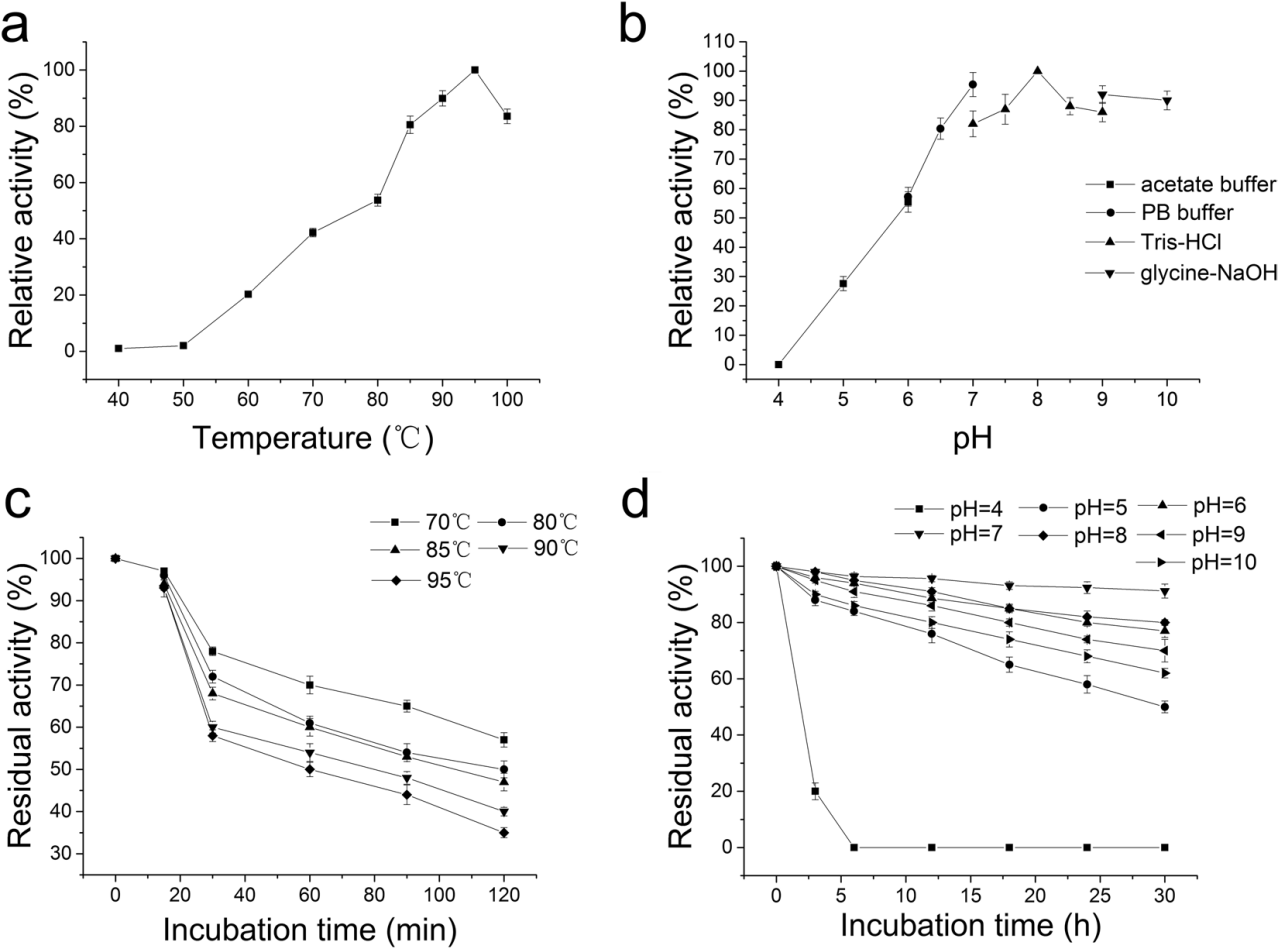
Effects of temperature and pH on L-asparaginase activity. (**a**) The optimal temperature was determined by assaying the activity from 40–100 °C in 0.05 M Tris-HCl buffer (pH 8.0). (**b**) The influence of the pH on the enzyme activity was investigated by assaying the activity at various pH values at 95 °C. (**c**) The enzyme thermostability was determined by incubation at different temperatures for a specified time at pH 7.0, followed by assay of the residual activities. (**d**) The influence of pH on the enzyme stability was determined by incubating at different pH values at 4 °C for a specified time and assaying the residual activities.

The relative activity of the *P. yayanosii* CH1 L-asparaginase was determined at 40–100 °C (Fig. 1a). Additionally, the thermostability was assayed at both the working temperatures (70–95 °C) and storage temperatures (−20–37 °C) (Fig. 1c). At temperatures above 85 °C, the relative L-asparaginase activity was more than 80%, with maximal activity found at 95 °C (Fig. 1a). This result showed that the *P. yayanosii* CH1 L-asparaginase was thermophilic and that its optimum temperature was higher than that of other thermophilic enzymes (Supplementary Table 2). Compared with the L-asparaginases from mesophilic bacteria, the *P. yayanosii* CH1 thermophilic L-asparaginase exhibited higher enzyme activity and better thermal stability, although its specific activity was not as high as that of the L-asparaginases from other thermophilic bacteria. However, the thermostability of the *P. yayanosii* CH1 L-asparaginase was higher than that of some thermophilic bacteria, such as *Archaeoglobus fulgidus* and *Thermococcus zilligii*^12,30^. The enzyme exhibited high stability at both the working temperatures (70–95 °C) and storage temperatures (−20–37 °C). L-asparaginase also retained 57%, 50%, 47%, 40%, and 35% of its enzyme activity after 2 h of exposure to temperatures between 70, 80, 85, 90 and 95 °C respectively (Fig. 1c), with a half-life (t_(1/2, 85°C)_) of 105 min at 85 °C. After storage for one month at −20 and 4 °C, L-asparaginase displayed nearly unaltered activity, although there was a slight decrease in the activity of up to 90% after storage at 20 and 37 °C for the same period. Analysis of the secondary structure of L-asparaginase using circular dichroism (CD) spectroscopy in the 190–250 nm region (Supplementary Fig. 2) and calculating the α-helix, β-sheet, β-turn, and random coil proportions by the online server DICHROWEB (Supplementary Table 3) found that the L-asparaginase has abundant α-helical structure of approximately 42%. Since the secondary structure has reported as an important factor that affects protein thermostability and higher percentage of secondary structured residues known to enhance protein thermostability^31–33^, the abundant α-helical structure of the L-asparaginase probably contributed to the great thermostability of L-asparaginase.

Moreover, the enzyme exhibited higher activity under alkaline conditions than under acidic conditions, with a maximum activity observed at pH 8.0 (Fig. 1b). However, no significant difference was found in L-asparaginase activity between pH 7.0 and 10.0, although the enzyme displayed less than 57% relative activity at pH values below 6.0. Therefore, L-asparaginase was relatively unstable under acidic conditions and showed extremely decreased activity at pH 4.0, with no further activity after incubation for 6 h. In contrast, the enzyme was more stable under alkaline conditions, displaying approximately 75% activity after incubation at pH 6–10 for 30 h (Fig. 1d). The effects of the temperature and pH on L-asparaginase activity revealed that the enzyme could be used for high temperature alkaline industrial processes due to its stability in both the work and storage environments.

**Figure 2:**
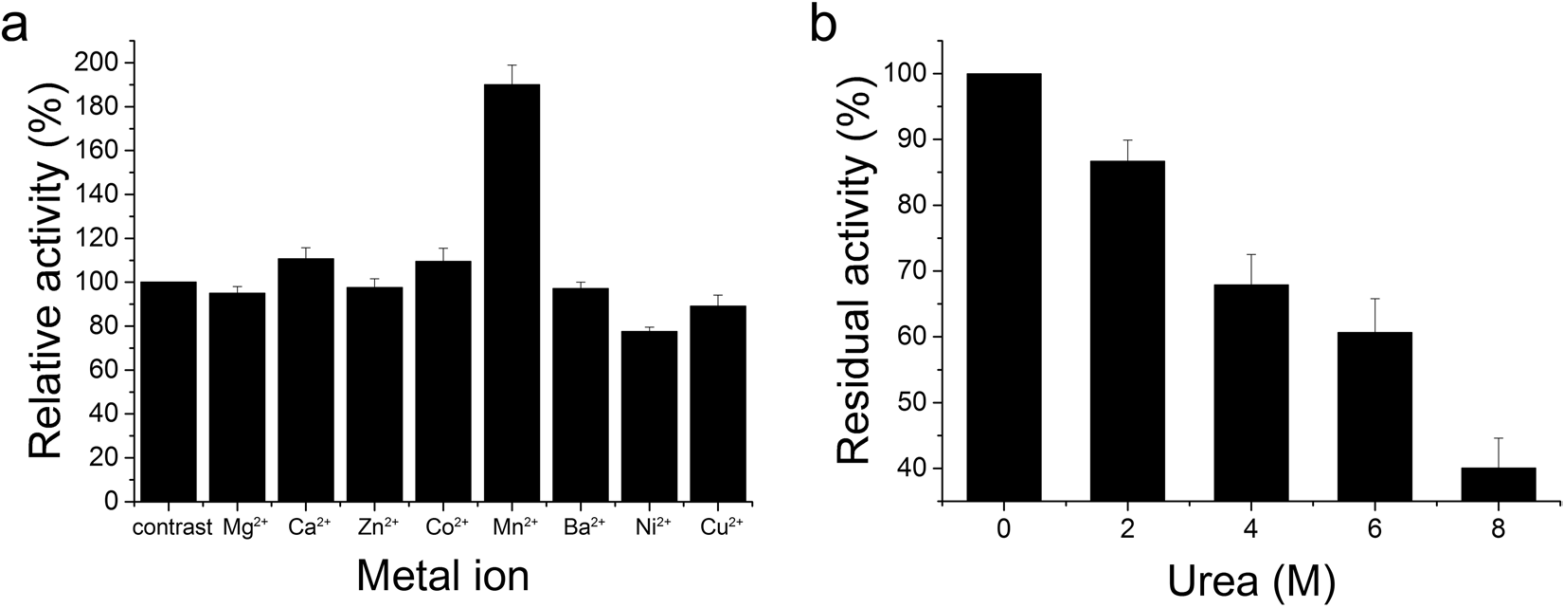
Effect of metal ions and urea. (**a**) Effects of 1 mM of different metal ions on L-asparaginase activity. (**b**) Effects of different urea concentrations on L-asparaginase activity.

Some divalent cations are essential cofactors for many catalytic reactions, and their presence may impact these reactions. Furthermore, some L-asparaginases from different sources are sensitive to urea^34,35^. Therefore, the effects of metal ions and urea on L-asparaginase were examined (Fig. 2). The results showed that L-asparaginase activity was strongly enhanced by Mn^2+^ (190%) and inhibited by Ni^2+^ (78%), whereas the addition of other metal ions (Mg^2+^, Ca^2+^, Zn^2+^, Co^2+^, Ba^2+^, and Cu^2+^) had little effect (less than 10%) on L-asparaginase activity (Fig. 2a). L-asparaginase was prone to urea denaturation since it displayed gradually reduced residual activities (86.7%, 67.9%, 60.7%, and 40.1%) after 2 h of incubation in 2, 4, 6, and 8 M urea, respectively (Fig. 2b).

Furthermore, the kinetics of L-asparaginase were determined as previously described in the Methods section. The recombinant hydrolysed L-asparagine enzyme had a Michaelis constant K_m_ value of 6.5 mM and a maximum reaction rate V_max_ of 2929 μM/min, which were relatively higher than those of mesophilic bacterial L-asparaginases but lower than those of thermophilic bacterial L-asparaginases^9,19,36^.

### Identification of variant with high L-asparaginase activity

To identify a high-activity thermophilic L-asparaginase variant, we developed a new robust screening method where cell disruption and enzyme activity assays were conducted simultaneously in 96-deep well plates. In this method, the cells were disrupted at high temperature, exposing the L-asparaginase, which was then reacted with Nessler’s reagent to produce a rufous dilute ammonia solution. The measured absorbance at 450 nm on a microplate reader showed the amount of ammonia released during the reaction. The amount of ammonia here was produced not only by the L-asparaginase activity but also by *B. subtilis* and L-asparagine self-hydrolysis. However, the amount of ammonia formed from *B. subtilis* and L-asparagine self-hydrolysis was nearly the same between the different wild-type strains and their mutants under the same conditions. Therefore, the difference in the amount of ammonia released was presumed to be due to L-asparaginase activity and thus was used to represent the strain L-asparaginase activity. In comparison to the strain activity of *B. subtilis* 168/pMA5-*pyasnase*, we screened positive variants.

**Figure 3:**
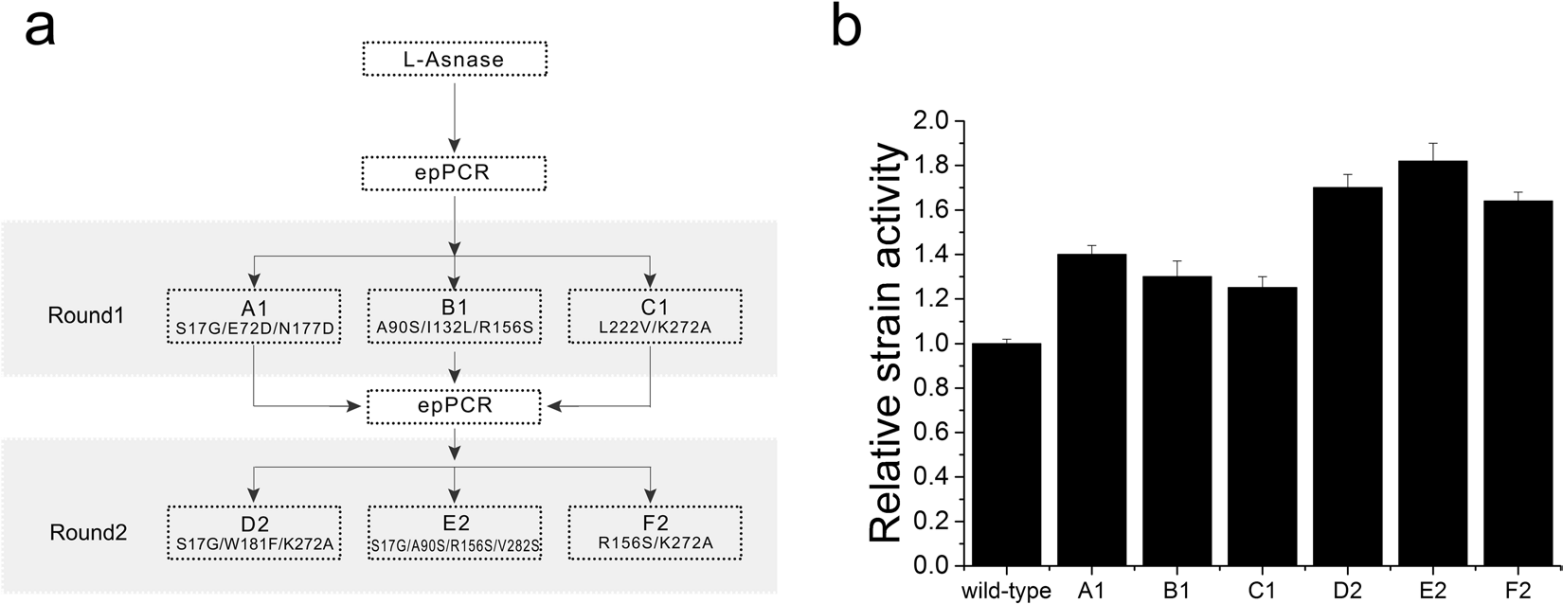
Screening for high-activity variants. (**a**) Schematic representation of the mutation and screening methods used to identify high-activity variants. (**b**) The strain activity of the wild-type and positive mutants.

**Figure 4:**
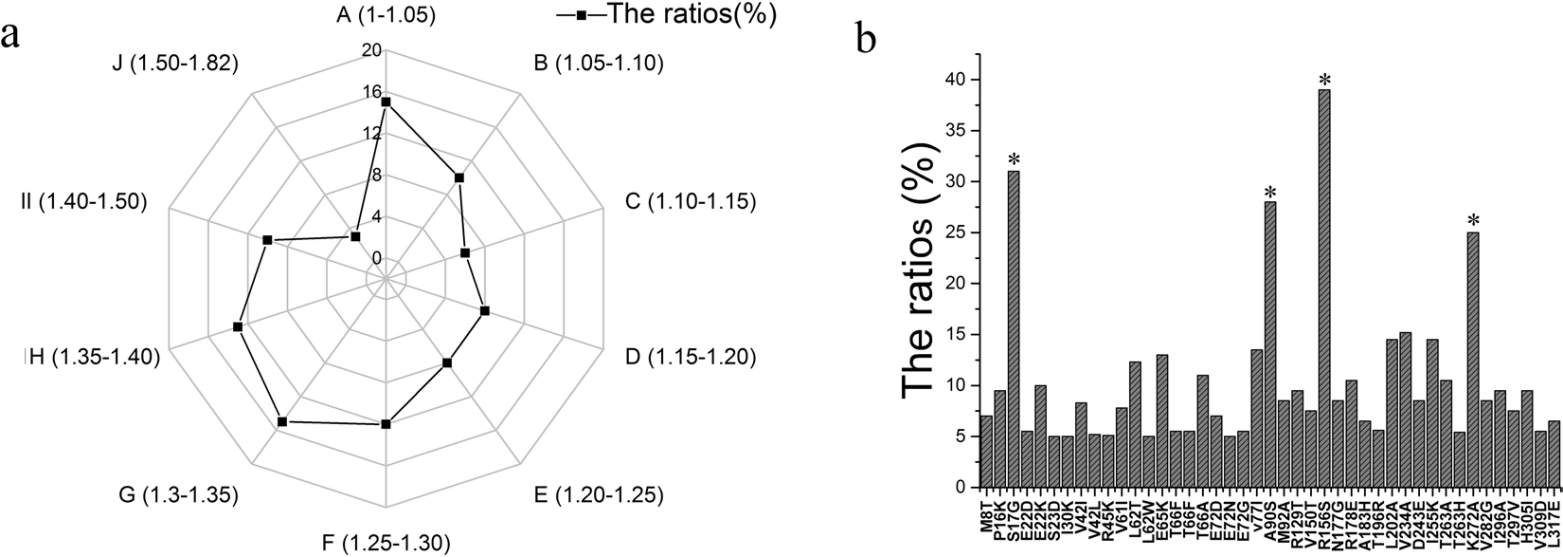
The range of relative strain activity and extent of amino acid residue mutations in positive variants. (**a**) The ratios of positive variants with different relative strain activity. A, B, C, D, E, F, G, H, I, J were the ratios of positive variants with 1 to 1.05, 1.05 to 1.10, 1.10 to 1.15, 1.15 to 1.20, 1.20 to 1.25, 1.25 to 1.30, 1.30 to 1.35, 1.35 to 1.40, 1.40 to 1.50, 1.50 to 1.82 fold strain activity than that of wild-type. (**b**) The ratios of positive variants with different amino acid residue mutations. The ratios of positive variants with different amino acid residues higher than 5% among the positive variants from two round of diversification and screening.

To obtain a mutagenesis library, two rounds of epPCR were performed (Fig. 3a). In every round, 3000 clones were screened. In the first round of screening, 213 positive variants were identified. Among these mutants, A1 (S17G/E72D/N177D), B1 (A90S/I132L/R156S), and C1 (L222V/K272A) were identified with higher L-asparaginase strain activity (Fig. 3b). These three variants were pooled and used as parents for the second round of diversification. Three brilliant positive variants were identified among the 568 positive variants found in the second round of screening. The mutants D2 (S17G/W181F/K272A), E2 (S17G/A90S/R156S/V282S), and F2 (R156S/K272A) showed higher L-asparaginase strain activity than the other mutants. Meanwhile, the relative strain activity of all positive variants from two round of diversification were determined (Fig. 4a). Compared with wild-type, the highest relative strain activity was 1.82 shown by mutant E2. Due to unavoidable errors and existing false positives in the positive variants, the ratio of relative strain activities within 1 to 1.10 folds was more than 20%. Besides that, most of the positive variants had relative strain activity of 1.25 to 1.40.

To validate the screened strains with high L-asparaginase activity, enzymatic characterization of the six positive mutants was also performed (Table 3). All six mutants showed higher specific activity than the wild-type strain. As shown in Fig. 3b and Table 3, the strain activities of A1, B1, C1, D2, E2, and F2 were 1.40, 1.30, 1.25, 1.70, 1.82, and 1.64-times that of the wild-type strain respectively. Additionally, the specific activities of A1, B1, C1, D2, E2, and F2 were 1.56, 1.45, 1.10, 1.81, 1.95, and 1.68-times those of the wild-type strain respectively. However, the strain activity was not solely consistent with the specific activity but instead revealed trends in specific activity. The strain activity and specific activity indicated that the screening method could effectively identify a high-activity mutant and reveal its activity in a semi-quantitative manner.

**Table 3:** The characteristics of L-asparaginase (wild-type) and its mutations.

Enzyme	Specific activity (U/mg)	Fold	t_(1/2,85°C)_ (min)	Optimum temperature (°C)	Optimum pH
Wild-type	1483.81 ± 63.61	1	105 ± 5	95	8.0
A1	2314.50 ± 93.12	1.56	105 ± 5	95	8.0
B1	2145.41 ± 78.36	1.45	140 ± 5	95	8.0
C1	1633.41 ± 83.15	1.10	100 ± 5	95	8.0
D2	2687.4 ± 84.11	1.81	110 ± 5	95	8.0
E2	2899.12 ± 97.35	1.95	150 ± 5	95	8.0
F2	2490.50 ± 65.44	1.68	100 ± 5	95	8.0
S17G	2556.25 ± 88.12	1.72	110 ± 5	95	8.0
A90S	1462.3 ± 75.33	0.99	150 ± 5	95	8.0
R156S	2414.33 ± 68.55	1.62	105 ± 5	95	8.0
K272A	1889.32 ± 59.9	1.27	100 ± 5	95	8.0
S17G/A90S/R156S/K272A	3108.43 ± 105.3	2.10	145 ± 5	95	8.0

The wild-type enzyme in this study was the *Pyrococcus yayanosii* CH1 L-asparaginase.

In this screening method, cell disruption and activity assays were simultaneously performed, which simplified the screening process and overcame the challenge of screening intracellular thermophilic enzymes. This method is anticipated to be valuable for high-throughput screening of high-activity thermophilic L-asparaginases and may be applicable for screening other thermophilic enzymes if a suitable colour-rendering method can be found.

### Analysis of the effect of L-asparaginase mutations

To find the most significant amino acid residue mutation to improve the relativity strain activity, we sequenced all 880 positive variants and determined the percentage of positive variants with different amino acid residue mutations higher than 5% among all positive variants (Fig. 4b). The percentage of positive variants with S17G, A90S, R156S or K272A amino acid residue mutation were 31%, 28%, 39% and 25%, respectively, which were much higher than the rates of the positive variants without these four residue mutations. Hence, the four mutations (S17G, A90S, R156S and K272A) were analysed, and single point mutations were introduced using site-directed mutagenesis. The enzymatic characterization of the single point mutations is shown in Table 3. The specific activities of S17G (2556 U/mg), R156S (2414.33 U/mg) and K272A (1889.32 U/mg) were higher than those of the wild-type enzyme (1483.81 U/mg), whereas the t_(1/2,85°C)_ of A90S (150 min) was longer than that of the wild-type (105 min). No significant difference was found in the other enzyme characteristics between the mutants and wild-type. The result indicated that the S17G, R156S and K272A mutants possessed beneficial specific activities, whereas A90S had improved thermostability.

To verify the effect of mutations on the enzyme, the secondary, tertiary and quaternary structures of L-asparaginase were analysed. Circular dichroism analysis of the mutant and wild-type enzymes revealed no significant alterations in the percentages of α-helices, β-sheets, β-turns and random coils (Supplementary Fig. 2, Supplementary Table 3), confirming that the increased specific activity and thermostability were not due to the secondary structure. Native-PAGE was used to verify whether the mutation altered the quaternary structure of the enzyme. The native-PAGE bands showed no significant differences in the quaternary structures of the mutant and wild-type enzymes (Supplementary Fig. 3). Thus no mutational alterations occurred.

**Figure 5:**
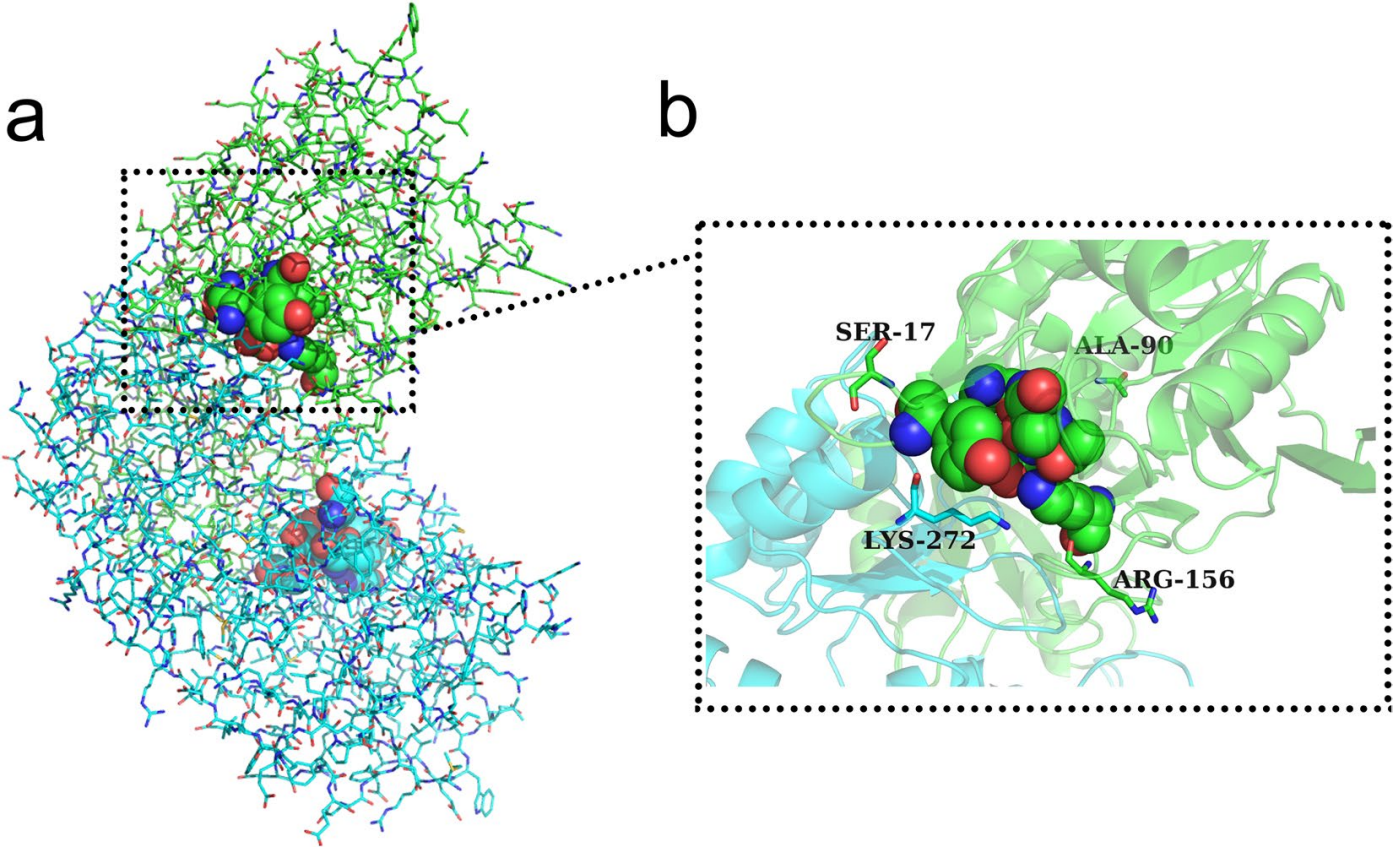
The tertiary structure of the *P. yayanosii* CH1 L-asparaginase. (**a**) Structural overview of the *P. yayanosii* CH1 L-asparaginase. The two subunits are shown with the green and blue lines. The residues located in the active domains are shown as balls. (**b**) The structure of the *P. yayanosii* CH1 L-asparaginase around the active domains. The S17, A90, R156 and K272 residues are shown as sticks.

**Figure 6:**
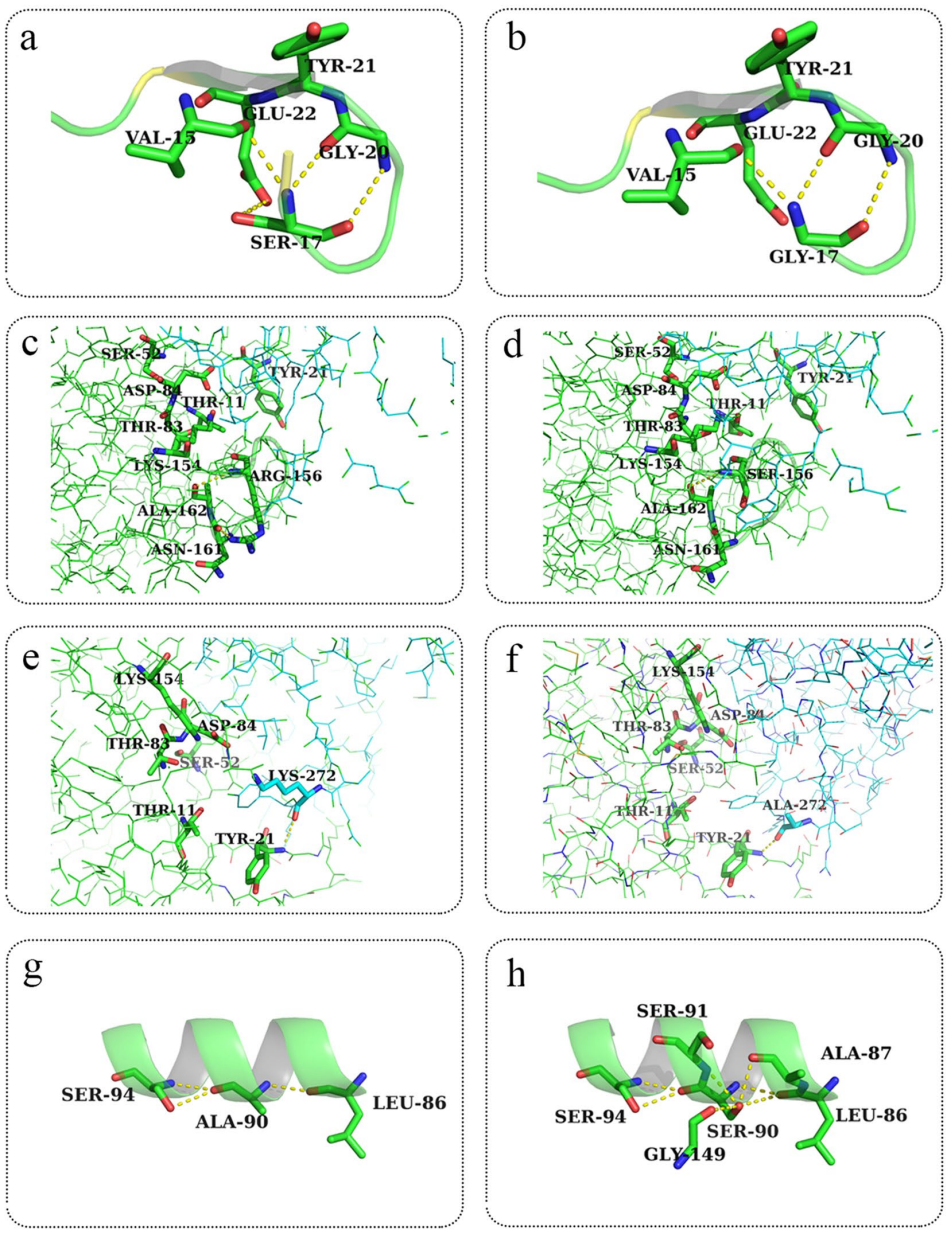
Tertiary structure of the wild-type and mutant enzymes. (**a**,**c**,**e**,**g**) are the tertiary structures around the S17, R156, K272, and A90 residues of the wild-type strain, respectively. (**b**,**d**,**f**,**h**) are the tertiary structures around the S17G, R156S, K272A, and A90S mutations, respectively.

Homologous modeling was used to explain the effect of mutations on the enzyme at tertiary structures. The structure from the wild-type L-asparaginase and its mutants were modelled using the crystal structure of *Pyrococcus furiosus* L-asparaginase (PDB ID: 4Q0M) as a template, with 62.35% identity. SWISS-MODEL showed that the global model quality estimate (GMQE) of this model was 0.85. The VERIFY_3D Server, which was used to check the resulting model, showed that 92.02% of the residues had an average 3D-1D score larger than 0.2, meaning that the model passed the quality check (≥80% 3D-1D scores larger than 0.2). Therefore, the protein structure model represented the accepted stereochemistry^27^. These models were used to explain the differences in thermostability and specific activity between L-asparaginase and its respective mutants in terms of the structure using PYMOL. Sequence alignment was used to identify the active domains. Amino acid residues involved in the catalytic mechanism of *E. coli* L-asparaginase (EcA) were identified previously^37^. The residues T12, Y25, S58, T89, D90 and K162 of EcA were crucial for the active site and were highly conserved in the *Pyrococcus yayanosii* CH1 L-asparaginase, corresponding to T11, Y21, S52 T83, D84 and K154, respectively (Supplementary Fig. 5). Based on this information, the molecular structure and active regions of *Pyrococcus yayanosii* CH1 L-asparaginase are shown in Fig. 5a. The L-asparaginase molecular structure was determined to be a homodimer, with each monomeric subunit consisting of 328 amino acid residues. Each monomer had an active region located at the interface of the other monomer. The mutated residues S17G, R156S, and K272A were located on different flexible loops, and A90S was located on an α-helix; additionally, the four mutated residues were located around the active domains in the structure space (Figs 5b and 6). Analysis of the molecular structure revealed that residue S17 formed two polar contacts with G20 and one polar contact with V15 and E22. The four residues with active site Y21 were in the same loop (Fig. 6a). S17 and G17 formed no polar contacts with E22. The deficiency of the polar contact with E22 may have contributed to the flexibility of the loop, thereby easing the binding of the substrate and the release of product (Fig. 6b). The S156 mutation had a smaller steric hindrance than R156, and the deficiency of the polar contact with N161 made the loop (residues 155–162) more flexible (Fig. 6c and d), which could have made substrate binding and release of the product occur more smoothly. The effect of the K272A mutation was similar to that of R156S, with both the K272 and A272 residues forming polar contacts with Y21, although A272 had a smaller steric effect than K272 (Fig. 6e and f). During substrate binding and product release with L- asparaginase, the opening and closing of the loop lid around activity site are absolute prerequisite^38,39^. An incompact structure around activity site may lead to free domains are to reorient spatially in a conformation state making it more accessible to the substrate, thereby increasing the specific activity^40^. S17G, R156S and K272A were located on the loops around the activity site. The deficiency of polar contact or smaller steric hindrance may have made binding of the substrate easier and product release smoother than in the wild-type. This explains the specific activity was increased after mutations. The α-helix and the number of hydrogen bond or salt bridge might have contributed to the protein stability^31,41,42^. The ninetieth residue of L-asparaginase was found in the core of the α-helix (86–96). Further, compare with A90 formed one polar contact with L86 and two polar contacts with S94 (Fig. 6g), S90 formed one polar contact with A87, S91, G149 and formed two polar contacts with L86 and S94 (Fig. 6h). The additional polar contacts may have contributed to the stability of the spatial structure around S90, thereby improving the thermostability of L-asparaginase. The results indicated that the improvement in the activity and thermostability was due to the alteration of the tertiary structures of the mutants.

### Construction of combinatorial mutant

To obtain L-asparaginase mutation with ideal properties, a combinatorial mutant (S17G/A90S/R156S/K272A) was constructed by Site-directed mutagenesis and showed high activity (3108 U/mg) and thermostability (t_(1/2,85)_ of 145 min). After 24 h of cultivation with basal medium in a 100-mL shake flask, the recombinant strain with the combinational mutations (*B. subtilis* 168/pMA5-S17G/A90S/R156S/K272A) showed extracellular and intracellular L-asparaginase activities of 48.21 and 129.95 U/mL, respectively. The L-asparaginase activity yield of *B. subtilis* 168/pMA5-S17G/A90S/R156S/K272A was 2 times higher than that of *B. subtilis* 168/pMA5-*pyasnase* in a shake flask. The improved yield may be due to the increased specific activity.

### Optimization of the medium and fed-batch culture

The recombinant *B. subtilis*168/pMA5-S17G/A90S/R156S/K272A strain was used for fermentation. Different sources and concentrations of carbon, nitrogen, inorganic nitrogen and corn steep liquor were used in the one-factor-at-a-time approach, and an orthogonal array design method was used to assess the biomass and L-asparaginase activity.

After optimization of the medium (Supplementary Fig. 5), 42 g/L of glycerine, 25 g/L of yeast, 1.5 g/L of NH_4_Cl and 15 g/L of corn steep liquor were found to be most suitable for biomass and L-asparaginase production. The orthogonal array method with four factors and four levels was used to analyse the effect of yeast, glycerine, NH_4_Cl and corn steep liquor on L-asparaginase production (Supplementary Table 4). The order of the factor effects on L-asparaginase production was as follows: corn steep liquor > yeast > glycerin > NH_4_Cl. A total of 35 g/L of yeast, 47 g/L of glycerine, 1.5 g/L of NH_4_Cl and 15 g/L of corn steep liquor was most suitable for L-asparaginase production. After using the one-factor-at-a-time and orthogonal array design methods, the optimal medium composition was determined to be as follows (g/L): glycerine, 47; yeast, 35; NH_4_Cl, 1.5; corn steep liquor, 15; K_2_HPO_4_, 2.612; KH_2_PO_4_, 2.041; MgSO_4_·7H_2_O, 1.845; NaCl, 5; and L-Asn, 1.

**Figure 7:**
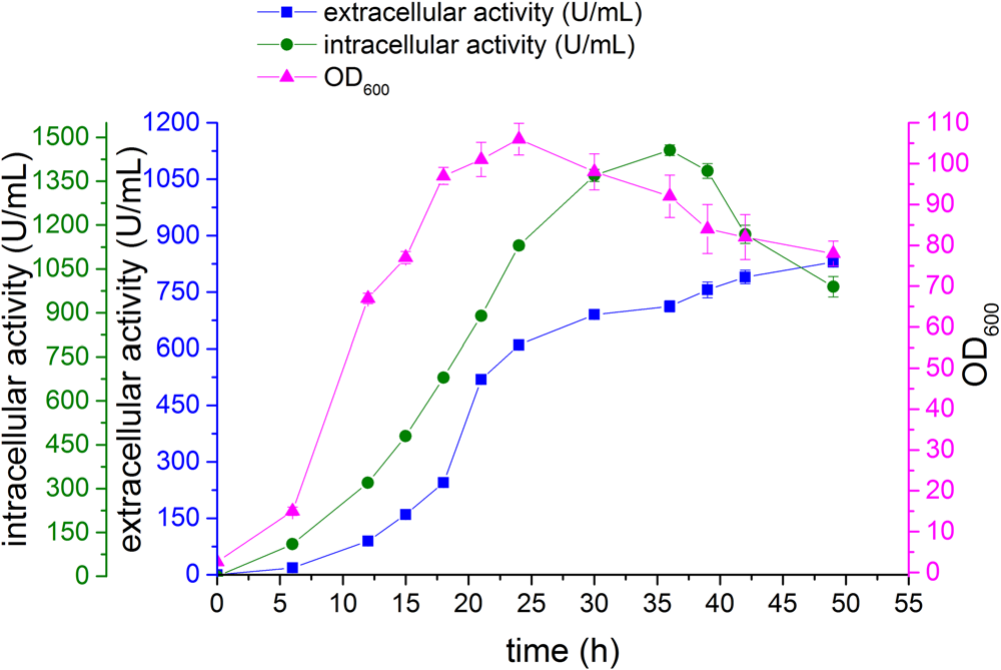
Fermentation of *B. subtilis* 168/pMA5- S17G/A90S/R156S/K272A in 5-L fermentation. Signal denotes the biomass (filled triangles), extracellular L-asparaginase activity (filled squares), and intracellular L-asparaginase activity (filled circles). The error bars are based on three biologically independent experiments.

L-asparaginase was fermented using pH-stat fed-batch operation strategies (Fig. 7). After 6 h of *B. subtilis* 168/pMA5-*pyasnase* fermentation (logarithmic phase), the L-asparaginase activity and biomass increased rapidly. The first feeding was initiated 15 hours into the fermentation, followed by an increase in the pH of the fermentation medium. After 20 h of fermentation, the L-asparaginase activity increased gradually, and autolysis occurred. After 36 h of fermentation, the maximum L-asparaginase activity of 2168 U/mL was obtained, and the intracellular and extracellular activities were 1456 U/mL and 712 U/mL, respectively. After 36 h of fermentation, the intracellular activity rapidly decreased and the extracellular activity increased due to autolysis of the cell.

In previous reports, the highest yield of L-asparaginase activity was 870 U/mL; this activity was achieved when *E. coli* DE3 was used to overexpress *E. coli* L-asparaginase and fermentation was performed in a 2-L fermenter. In addition to its high specific activity, the *P. yayanosii* CH1 L-asparaginase mutant had higher thermostability than the *E. coli* L-asparaginase and hence was resistant to temperature degradation during fermentation. These factors led to the high fermentation yield obtained with the *Pyrococcus yayanosii* CH1 L-asparaginase.

## Conclusion

A novel thermostable L-asparaginase from *P. yayanosii* CH1 was expressed in *B. subtilis* 168. We also developed a robust new method for screening and identifying high-activity mutants from a mutagenesis library via simultaneous high temperature cell disruption and enzyme activity assays. Using this method, four key mutants with improved activity and thermostability were identified. The specific activity of the combinatorial mutant S17G/A90S/R156S/K272A was 2.1 times greater than that of the wild-type L-asparaginase. Furthermore, fermentation of the mutant yielded L-asparaginase activity of 2168 U/mL, which was the highest reported activity to date, showing high potential for large-scale industrial production of L-asparaginase.

## Electronic supplementary material

Supplemental
