# Supplementary material for: Simultaneous cell disruption and semi-quantitative activity assays for high-throughput screening of thermostable L-asparaginases
Source: Sci Rep. 2018 May 21;8:7915. doi: 10.1038/s41598-018-26241-7 (PMC5962637; doi:10.1038/s41598-018-26241-7)
Supplement: Supplementary file 1 — Supplemental [file 41598_2018_26241_MOESM1_ESM.pdf]

Simultaneous cell disruption and semi-quantitative activity assays for high-throughput screening of thermostable L-asparaginases

Xu Li[a], Xian Zhang[a]*, Shuqin Xu[a], Hengwei Zhang[a], Meijuan Xu[a], Taowei Yang[a], Li Wang[b], Haifeng Qian[b], Huiling Zhang[c], Haitian Fang[c], Tolbert Osire[a], Zhiming Rao[a]*, Shangtian Yang[d]

[a] The Key Laboratory of Industrial Biotechnology, Ministry of Education, School of Biotechnology, Jiangnan University, Wuxi 214122, China

[b] School of Food Science and Technology, Jiangnan University, Wuxi 214122, China

[c] School of Agriculture Ningxia University, Yinchuan 750021, China

[d] Department of Chemical and Biomolecular Engineering, The Ohio State University, Columbus, OH 43210, USA

*Corresponding author at: The Key Laboratory of Industrial Biotechnology of Ministry of Education, School of Biotechnology, Jiangnan University, 1800 Lihu Road, Wuxi, Jiangsu 214122, China.

Tel: +86-510-85916881; fax: +86 510 85918516.

Email address: raozhm@jiangnan.edu.cn (Z. Rao).

Supplementary data

Supplementary table 1. The optimized sequence of *P. yayanosii* CH1 L- asparaginase gene for *Bacillus subtilis.*

sequences
atgagactgctgatcctgggaatgggaggaacaatcgcaagtgtgccttcagaagagggatacgaatcatcactgtctgtggaggagatc<br>ctgagacttgcaggacttgagctgaagtgggaagttgaggctagagatctgctgaacatcgattctacgttgatccagcctgaggattgggt<br>tctgctggctgaaacagtattcgaggcattcgaggaatttgacggagtggtaataacccacggtacagacacgctcgcttacacagcttcg<br>atgcttagctttatggtgagaaaccctcctgtgcctatcgtactcacgggagcaatgaggcctattacagagccaggttccgatgcaccaag<br>gaacttatggacagctttgagatttgctatcgaaggagtgccaggagtttacgtggcctttatggataaggtcatgctcggagtgagagtaag<br>caaggtccgtgcagttggtcttaacgcctttcaaagcattaattatccagacatagcctatgtcaagggcaatcgtattcattggaatgccaaa<br>ccgccgaaactcgaaggcgaaccggtgctcgacacgcgacatgaaccgcgtgttcttgtattgcgacttgttccgggtatggaaggcgat<br>gtacttgaagcggccttagaattgggttatcgcggtattgtccttgaaggctatggggtgggcgggattccgtatcgtggccgcgatttgctt<br>gatgttgttcggcgggttgcgactgaaattccggttgtaatgactacacaaacattatatgacggcgttgacttgaccaaatacaaagtcggc<br>cggaaagcgttagaagtcggcgtcattccggcgggggatatgactaaagaagcgaccattacgaaattaatgtggatattaggccatacg<br>cgcgatgtcggggaagtccggcgcttaatgttaaccaatatggtcggcgaaattgggaaatccgcgtaa

Supplementary table 2. The character of L-asparaginases from different sources. NR, not reported. The V_{max} of *Pectobacterium carotovorum* MTCC 1428 L-Asnase was 4.45U/ug.

Strains	GenBank accession No.	Specific activity (U/mg)	Optimum temperature (℃)	Optimum pH	V_{max} (uM/min)	Km (mM)	Reference
Pyrococcus yayanosii CH1	WP_013906452.1	1483	95	8	2929	6.5	This study
E.coli	WP_000394140.1	105.2	37	7.5	NR	0.03	Maggi et al., 2017
B. subtilis B11-06	AGT62618.1	92.45	40	7.5	77.51	0.43	Jia et al., 2013
T.gammatolerans EJ3	WP_015859055.1	7622	85	8.5	NR	10	Zuo et al., 2014
T. zilligii AN1 TziAN1_1	WP_010478656.1	5526	90	8.5	NR	6.02	Zuo et al., 2015
Thermococcus kodakaraensis KOD1	WP_011250607.1	2350	85	9.5	3300	5.5	Chohan & Rashid, 2013
Pyrococcus furiosus DSM 3638	WP_011013191.1	550	80	9	NR	12	Bansal et al., 2010
Pectobacterium carotovorum MTCC 1428	AFA36648.1	153.25	30	6.5		0.657	Kumar et al., 2011
vibrio succinogenes	WP_011138590.1	202	37	7.3	NR	0.478	Distasio et al., 1976

Supplementary table 3. The percentage of secondary structure in L-Asnase and its mutations.

This results were calculated by Online server DICHROWEB.

	α-helix(%)	β-sheet(%)	β-turn(%)	random coil(%)
L-Asnase	42.3	15.4	17.2	26.0
S17G	42.1	15.3	17.4	25.8
A90S	42.3	15.2	17.0	26.1
R156S	42.2	15.1	17.1	26.2
K272A	42.1	15.5	17.2	25.9

Supplementary table 4. The orthogonal experiments.

Factor	yeast（g/L）	glycerin（g/L）	NH_4Cl(g/L)	corn steep liquor（g/L）	activity（U/mL）
Test 1	20	37	1	12	114.88
Test 2	20	42	1.5	15	128.7
Test 3	20	47	2	18	98.45
Test 4	20	52	2.5	21	86.3
Test 5	25	37	1.5	18	105.3
Test 6	25	42	1	21	90.2
Test7	25	47	2	12	134.95
Test 8	25	52	2.5	15	129.45
Test 9	30	37	2	21	96.3
Test 10	30	42	2.5	18	107.5
Test 11	30	47	1	15	149.67
Test 12	30	52	1.5	12	141.18
Test 13	35	37	2.5	15	134.18
Test 14	35	42	2	12	138
Test 15	35	47	1.5	21	108.3
Test 16	35	52	1	18	119.2
average value 1	107.08	112.66	118.48	131.10	
average value 2	114.98	116.10	120.87	136.65	
average value 3	123.66	121.70	117.93	107.62	
average value 4	124.93	120.18	113.35	95.28	
range	17.85	9.04	7.52	35.82	

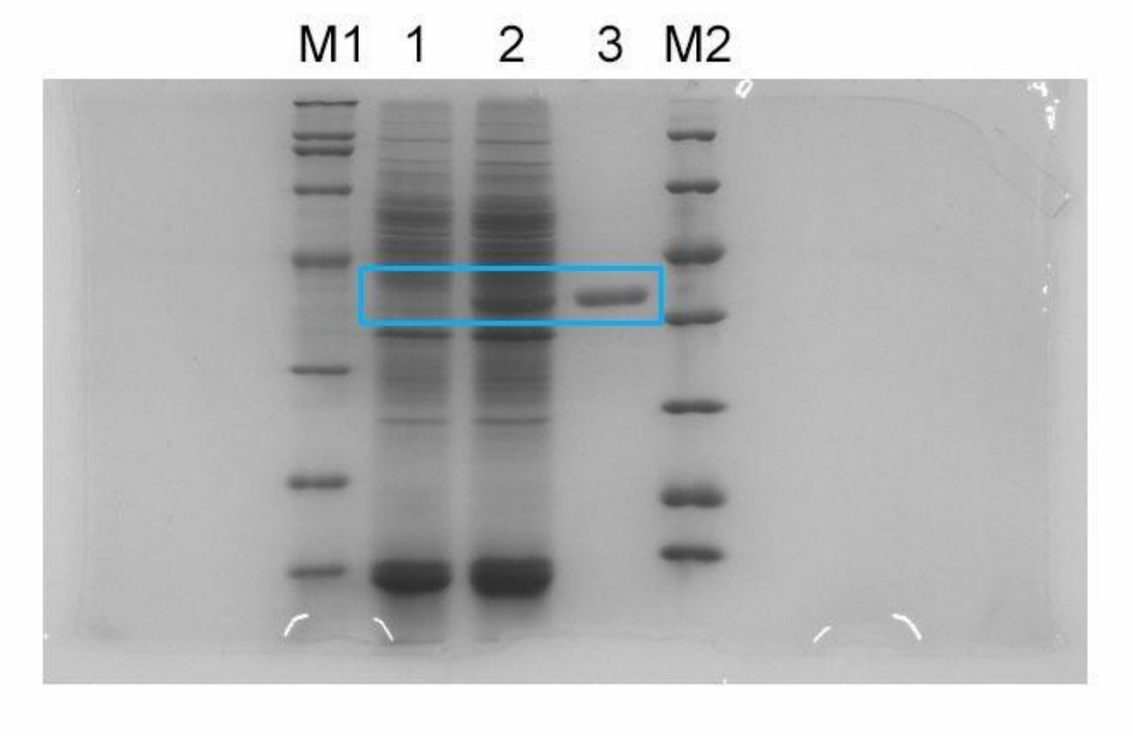

Supplementary figure 1. SDS-PAGE analysis of L-asparaginase from *B. subtilis* 168/pMA5-*pyasnase*. Lane M1, Premixed Protein Marker(Broad); Lane 1, *B. subtilis* 168/pMA5 cell extract; Lane 2, crude enzyme of *B. subtilis* 168/pMA5- *pyasnase*; Lane 3, the purified L- asparaginase; Lane M2, Unstained Protein Molecular Weight Marker.

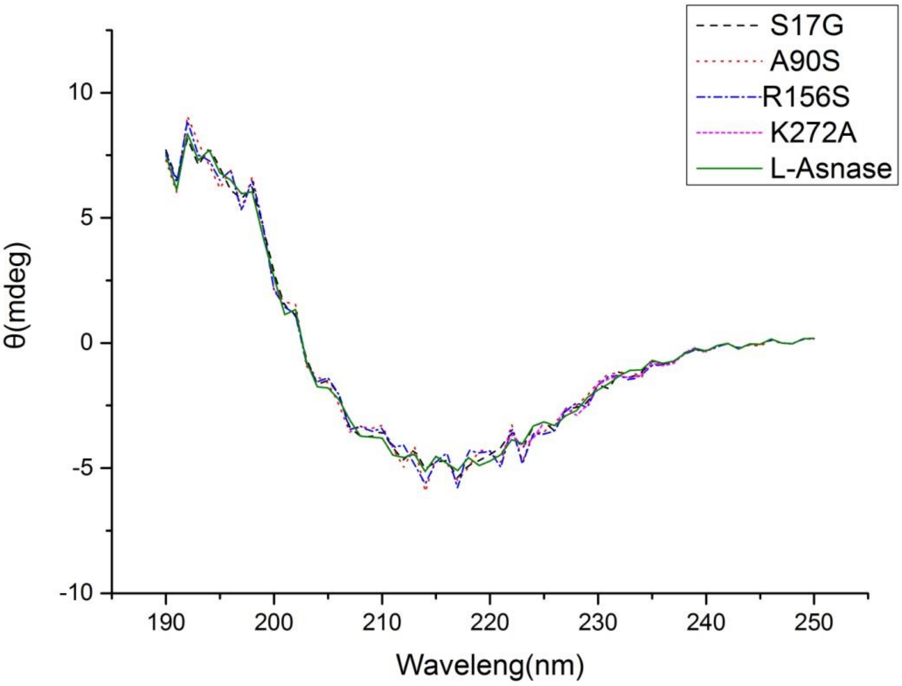

Supplementary figure 2. CD curves of *P. yayanosii* CH1 L-Asnase and its single mutations.

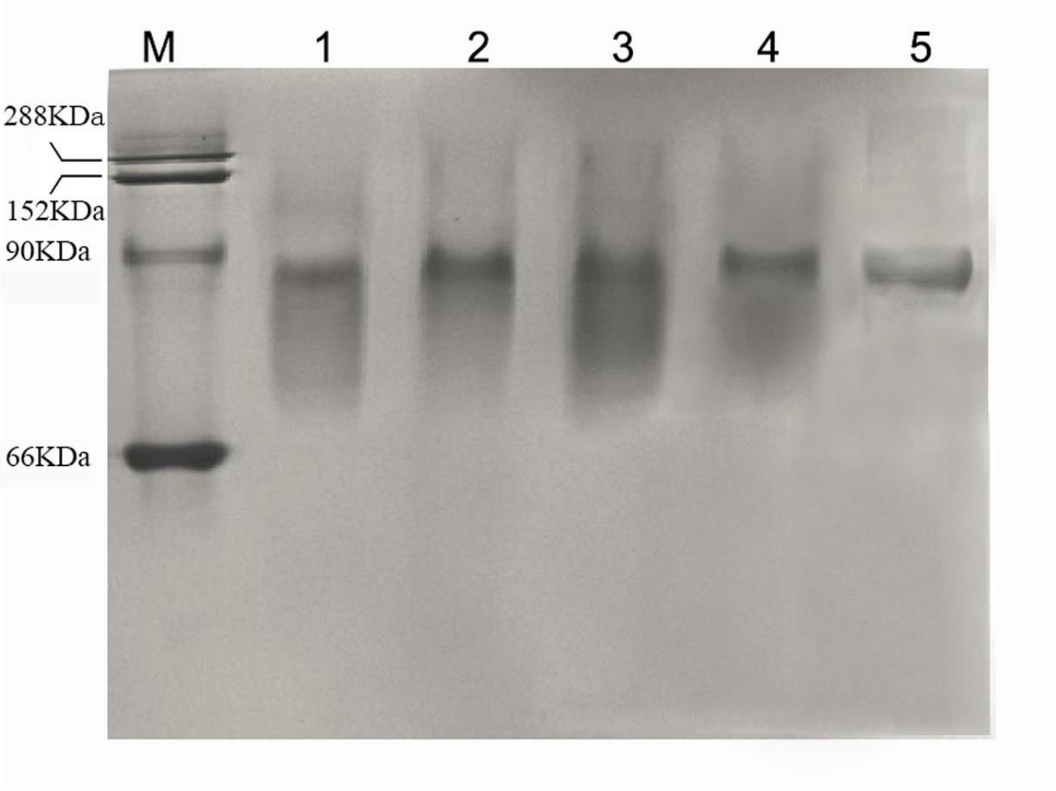

Supplementary figure 3. Native-PAGE analysis of *P. yayanosii* CH1 L-Asnase and its single mutations. Lane M: Marker. the marker was prepared by our lab. From top to bottom, the proteins, which were represented by the bands, were *bacillus cereus* Leucine Dehydrogenase(288kDa), *Bacillus subtilis* L-asparaginase II (152kDa), *Staphylococcus aureus* lactic dehydrogenase(90kDa), bovine serum albumin(66kDa). Lane 1: *P. yayanosii* CH1 L- asparaginase. Lane 2: The mutation S17G. Lane 3: The mutation A90S. Lane 4: The mutation R156S. Lane 5: The mutation K272A.

```
1.seq   LPNITILATGGTIAGGGDSATKSNYTVGKVGVENLVNAVPQLKDIANVKGEQVVNIGSQD      60
2.seq   .MRLLILGMGGTIAS...VPSEEGYES.SLSVEEILR.LAGIELKWEVEARDLLNIDSTL      54
Consensus    il  ggtia          y      ve        l     v      ni s

1.seq   MNDNVWLTLAKKINTDCDKTDGFVITHGTDTMEETAYFLDLTVKCD.KPVVMVGAMRPST     119
2.seq   IQPEDWVLLAETVFEAFEEFDGVVITHGTDTLAYTASMLSFMVRNPPVPIVLTGAMRPIT     114
Consensus    w  la          dg vithgtdt   ta  l   v     p v  gamrp t

1.seq   SMSADGPFNLYNAVVTAADKASANRGVLVVMNDTVLDGRDVTKTNTTDVATFKSVNYGPL     179
2.seq   EPGSDAPRNLWTALRFAIEGVP...GVYVAFMDKVMLGVRVSKVRAVGLNAFQSINYPDI     171
Consensus   d p nl  a   a        gv v   d v  g  v k        f s ny

1.seq   GYIHNGKIDYQRTPARKHTSDTPFDVSKLNELPKVGIVYNYANASDLPAKALVDAGYDGI     239
2.seq   AYVKGNRIHWNAKPP.KLEGEPVLDT...RHEPRVLVLRLVPGMEGDVLEAALELGYRGI     227
Consensusy     i     p  k       d       p v               a    gy gi

1.seq   VSAGVGNGNL.YKS..VFDTLATAAKTGTAVVRSSRVPTGATTQDAEVD..DAKYGFVAS     294
2.seq   VLEGYGVGGIPYRGRDLLDVVRRVATEIPVVMTTQTLYDGVDLTKYKVGRKALEVGVIPA     287
Consensuv  g g g   y      d     a     v        g       v       g

1.seq   GTLNPQKARVLLQLALTQTKDPQQIQQIFNQY........                         326
2.seq   GDMTKEATITKLMWILGHTRDVGEVRRLMLTNMVGEIGKS                         327
Consensug          l   l  t d
```

Supplementary figure 4 Sequence alignments of *E.coil* L-asparaginase and *Pyrococcus yayanosii* CH1 L-asparaginase. 1.seq: Sequence of *E.coil* L-asparaginase (WP_000394140.1); 2.seq: Sequence of *Pyrococcus yayanosii* CH1 L-asparaginase(WP_013906452).

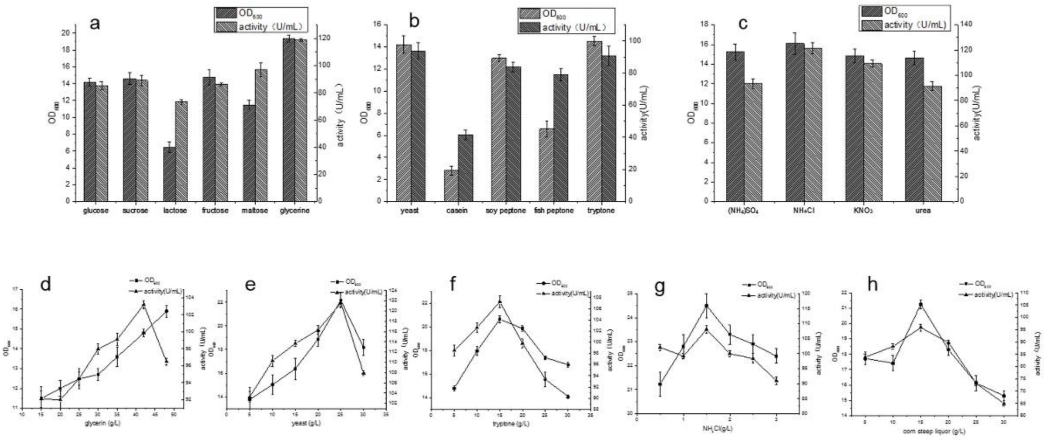

Supplementary figure 5. The effect of carbon, nitrogen, inorganic nitrogen source and corn steep liquor on the biomass and L-asparaginase activity. a) The effect of different kind carbon source with same concentration (35g/L) on the biomass and L-asparaginase activity. b) The effect of different kind nitrogen source with same concentration (15g/L) on the biomass and L-asparaginase. c) The effect of different kind inorganic nitrogen source with same concentration (0.8g/L) on the biomass and L-asparaginase. d) The effect of glycerine at different concentration (15, 20, 25, 30, 35, 42, 48g/L) on the biomass and L-asparaginase. e) The effect of yeast at different concentration(5-30g/L) on the biomass and L-asparaginase. f) The effect of tryptone at different concentration(5-30g/L) on the biomass and L-asparaginase. g) The effect of NH_4Cl at different concentration(0.5-3g/L) on the biomass and L-asparaginase. h) The effect of corn steep liquor at different concentration (5-30g/L) on the biomass and L-asparaginase.
